# Lymphangiome kystique cervico-thoracique: à propos d’un cas

**DOI:** 10.11604/pamj.2016.25.189.9363

**Published:** 2016-11-24

**Authors:** Karim Nadour, Mountassir Moujahid

**Affiliations:** 1 Service d’ORL et CCF, Hôpital Militaire My Ismail, CHU Fès, Maroc; 2 Service de Chirurgie Générale, Hôpital Militaire de Marrakech, CHU Marrakech, Maroc

**Keywords:** Lymphangiome kystique, cou, thorax, Cystic lymphangioma, neck, chest

## Abstract

Les lymphangiomes kystiques cervico-thoraciques sont des tumeurs bénignes rares, ils proviendraient d'une séquestration du sac lymphatique embryonnaire qui se remplirait progressivement de liquide lymphatique. Le diagnostic est évoqué par la clinique (tuméfaction latéro-cervicale) et l'imagerie (échographie et tomodensitométrie), puis confirmé par l'histologie après la chirurgie qui constitue la base du traitement. Nous rapportons un cas de lymphangiome kystique cervico-thoracique avec une revue de la littérature.

## Abstract (en)

Cervicothoracic cystic lymphangiomas are rare benign tumors developing from sequestration of embryonic lymph sac which is gradually filled with lymph fluid. The diagnosis is based on clinical signs (laterocervical swelling) and imaging (ultrasound and CT scan), then confirmed by histology after surgery which constitutes the basis of treatment. We here report a case of cervicothoracic cystic lymphangioma and review of the literature.

## Introduction

Les lymphangiomes kystiques cervico-thoraciques sont rares. Leur diagnostic est évoqué par l'existence d'une masse cervico-thoracique. L'imagerie médicale permet d'évoquer le diagnostic, mais seul l'examen anatomopathologique le confirme. Le traitement est essentiellement chirurgical. Nous rapportons un cas de lymphangiome kystique cervico-thoracique avec une revue de la littérature.

## Patient et observation

**Figure 1:**
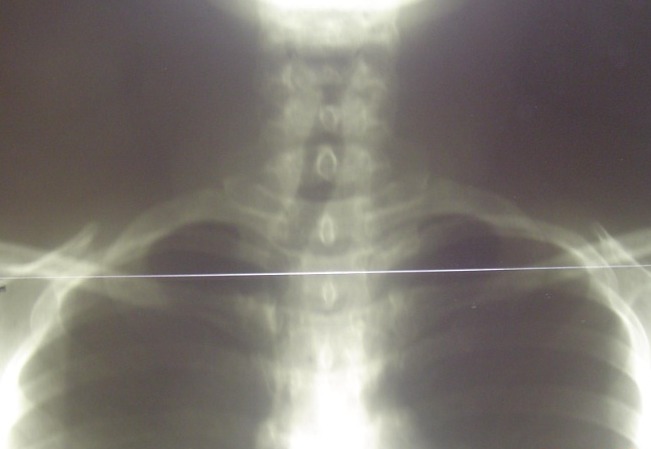
Radiographie pulmonaire de face montrant le refoulement de la trachée en dehors et à droite par cette masse latérocervicale gauche

**Figure 2:**
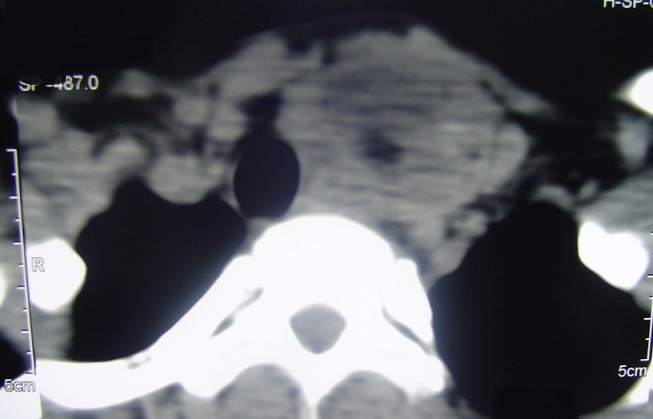
Scanner cervicothoracique en coupe axiale montrant la masse cervicomédiastinale supérieure antéro latérale gauche

**Figure 3:**
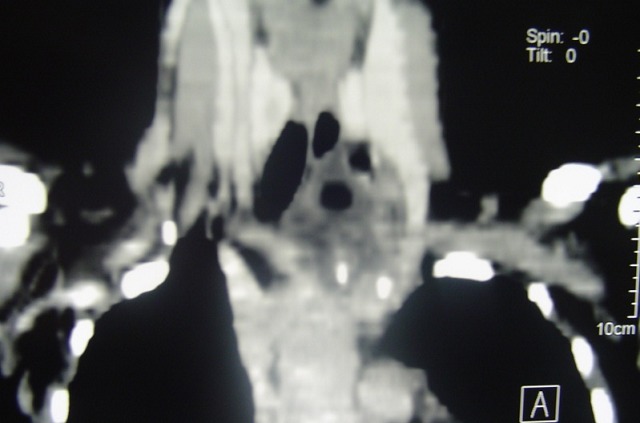
Coupe sagittale montrant cette masse hypodense et son prolongement intra-thoracique

**Figure 4:**
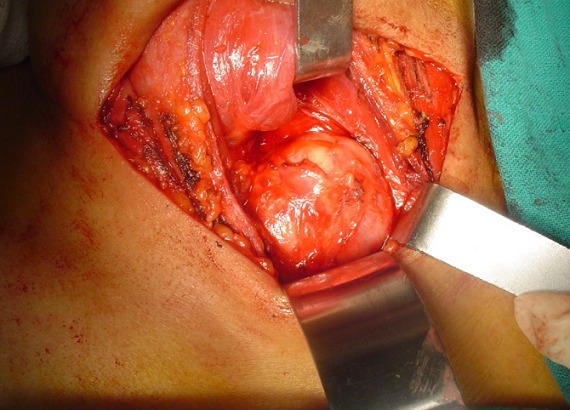
Vue opératoire montrant l’exérèse en totalité de la masse tumorale

**Figure 5:**
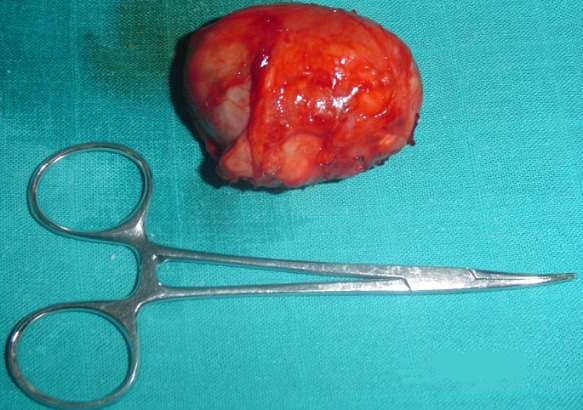
Pièce opératoire de lymphangiome kystique cervico thoracique

Patiente âgée de 50 ans sans antécédents pathologiques notables admise en mai 2007 pour prise en charge d'une masse cervico-thoracique gauche évoluant depuis six mois. Sur la radiographie pulmonaire, cette masse entrainait une déviation trachéale à droite (Figure 1). L'échographie cervicale a objectivé une masse liquidienne hypoéchogéne paramédiane gauche mesurant 70 mm de long en contact étroit avec le pole inférieur du lobe thyroïdien gauche, cette masse se prolongeait dans le thorax. Le scanner cervico thoracique a montré une masse cervico-médiastinale supérieure antérolatérale gauche de composante liquidienne faisant environ 7cm de hauteur, comprimant les structures cervico-médiastinales en regard notamment la veine jugulaire qui est refoulée vers l'extérieur, la trachée refoulée en dehors et à droite et le pole inférieur du lobe gauche de la glande thyroïde qu'elle soulève en haut (Figure 2 et Figure 3). La patiente a bénéficié d'une cervicotomie sus sternale gauche (Figure 4 et Figure 5), qui a permis l'exérèse complète de cette masse. Les suites post opératoires ont été simples et le compte rendu anatomopathologique était en faveur d'un lymphangiome kystique. Les contrôles ultérieurs n'ont pas objectivé de récidive après un recul de 6 ans.

## Discussion

Les lymphangiomes sont des tumeurs bénignes rares. Trois types de lymphangiomes peuvent être distingués : les lymphangiomes capillaires comprenant les petits vaisseaux à lumière étroite, les lymphangiomes caverneux à lumière dilatée, anfractueuse et inter communicante et les lymphangiomes kystiques ou hygroma kystique présentant de larges cavités confluentes remplies de liquide jaune clair [1]. Deux théories pathogéniques sont évoquées dans la littérature [2]: la théorie mécanique expliquant la survenue de ces kystes suite à une obstruction ou une contusion lymphatique ; mais cette théorie est rarement confirmée par l'histoire clinique, et la théorie congénitale la plus admise actuellement. Le lymphangiome proviendrait d'une séquestration [3] du sac lymphatique embryonnaire qui se remplirait progressivement de liquide lymphatique. L'échec de l'établissement d'anastomose entre les vaisseaux normaux et pathologiques, et l'accumulation de liquide lymphatique, seraient responsables de la genèse de cette lésion [4]. Les lymphangiomes kystiques sont le plus souvent isolés, exceptionnellement diffus réalisant la lymphangiomatose [3]. A part le cerveau, les lymphangiomes peuvent être trouvés dans tout le corps, ils peuvent se localiser dans l'abdomen, la cavité buccale, le médiastin, la région axillaire et inguinale mais les lymphangiomes touchent en grande partie la région cervicale dans environ 75% des cas [1, 3]. La localisation cervicale se rencontre plus dans l'enfance : 90% avant l'âge de 20 ans, mais peut être découverte à tout âge de la vie en raison de la latence d'évolution [2, 4]. D'autres localisations ont été également citées : la localisation rétro péritonéale, splénique, colique [4], musculaire [5] et même au niveau du cordon spermatique La localisation cervico thoracique de notre observation ferait avancer l'hypothèse selon laquelle il résulterait de la migration d'éléments lymphatiques initialement séquestrés à l'étage cervical et qui aurait suivi dans leur déplacement vers le bas d'autres éléments migrateurs comme le thymus, les bourgeons bronchiques, le cœur ou le péricarde [1, 5]. Ce qui laisse penser que les lymphangiomes kystiques cervicaux ont une extension médiastinale.

La symptomatologie clinique est fonction de la taille de la tumeur et de la topographie de la formation kystique. En dehors de la masse cervicale palpable, les lymphangiomes kystiques n'ont pas de spécificité clinique. Ainsi la circonstance de découverte des lymphangiomes kystiques cervico-thoraciques est parfois une symptomatologie révélatrice telle que la masse cervicale comme c'était le cas pour notre patiente; parfois une symptomatologie d'emprunt, mais dans 50% des cas, ils sont de découverte fortuite lors d'une radiographie pulmonaire. La radiographie standard montre une opacité de siège médiastinal antérieur ou postérieur dont l'aspect n'est pas spécifique. L'échographie montre un aspect hypoéchogène ou anéchogène, parfois avec un sédiment ou de fins échos internes et un renforcement postérieur d'échos [2, 6]. Le scanner montre une tumeur de faible densité liquidienne (10-36 UH) mais les cloisons ne sont parfois révélées qu'après injection du produit de contraste [2, 4]. L'imagerie par résonance magnétique semblerait être utile pour l'exploration de cette tumeur, mais s'avérerait moins performante que la tomodensitométrie en cas de complications [4]. Seule l'histologie permet d'avoir un diagnostic de certitude [1, 2]. Le traitement est essentiellement chirurgical permettant l'exérèse complète de la tumeur indispensable pour avoir une guérison complète. D'autres moyens thérapeutiques ont été essayés sans succès, comme la radiothérapie, le drainage par médiastinoscopie et la sclérose chimique par cyclophosphamide intra veineux [2, 6], ils sont surtout réservés aux tumeurs non résecable à cause de leur taille, de leur localisation ou à cause de l'état général du patient [6]. La vidéothoracoscopie, le drainage par médiastinoscopie ou par ponction scanno ou écho guidée n'assurent pas l'exérèse de la paroi du kyste source de récidive. La voie d'abord dépend de la localisation et des prolongements kystiques. La thoracotomie postéro latérale est préconisée par de nombreux auteurs pour la localisation médiastinale pure [2, 4, 6]. La localisation cervico-médiastinale peut amener à choisir une cervicotomie sus sternale associée parfois à une sternotomie médiane en fonction du prolongement endothoracique, des adhérences viscérales aux gros vaisseaux, aux structures nerveuses ou à la trachée. Dans notre cas nous avons réalisés une simple cervicotomie sus sternale qui nous a permis d'enlever la tumeur en sa totalité, la relative facilité de l'intervention repose sur le fait que la formation kystique était bien circonscrite de volume modéré avec un plan de clivage évident sans poussée inflammatoire et sans adhérences aux structures vasculaires permettant l'exérèse totale de la tumeur. Les suites opératoires sont généralement excellentes.

## Conclusion

Les lymphangiomes kystiques cervico-thoraciques sont rares. Ils se manifestent généralement par une masse latéro-cervicale basse isolée, une tomodensitométrie cervico-thoracique est souvent nécessaire pour aider au diagnostic mais surtout pour étudier le prolongement intra-thoracique et ses rapports avec les structures vasculaires, l'exérèse chirurgicale complète souvent par une cervicotomie est la base du traitement, les suites opératoires à court et à long terme sont souvent excellentes.
